# Prenatal Care Utilization for Mothers from Low-Income Areas of New Mexico, 1989–1999

**DOI:** 10.1371/journal.pone.0012809

**Published:** 2010-09-17

**Authors:** Michael A. Schillaci, Howard Waitzkin, E. Ann Carson, Sandra J. Romain

**Affiliations:** 1 Department of Social Sciences, University of Toronto Scarborough, Scarborough, Ontario, Canada; 2 Departments of Sociology, Internal Medicine, and Family & Community Medicine, University of New Mexico, Albuquerque, New Mexico, United States of America; 3 Intellica Corporation, San Antonio, Texas, United States of America; 4 Department of Anthropology, University of Toronto, Toronto, Ontario, Canada; Aga Khan University, Pakistan

## Abstract

**Background:**

Prenatal care is considered to be an important component of primary health care. Our study compared prenatal care utilization and rates of adverse birth outcomes for mothers from low- and higher-income areas of New Mexico between 1989 and 1999.

**Methodology/Principal Findings:**

Prenatal care indicators included the number of prenatal care visits and the first month of prenatal care. Birth outcome indicators included low birth weight, premature birth, and births linked with death certificates. The results of our study indicated that mothers from low-income areas started their prenatal care significantly later in their pregnancies between 1989 and 1999, and had significantly fewer prenatal visits between 1989 and 1997. For the most part, there were not significant differences in birth outcome indicators between income groupings.

**Conclusions/Significance:**

These findings suggest that while mothers from low-income areas received lower levels of prenatal care, they did not experience a higher level of adverse birth outcomes.

## Introduction

This retrospective research compared levels of prenatal care utilization and rates of adverse birth outcomes between mothers from low- and higher-income residential areas of New Mexico, a largely poor and rural state with high levels of poverty, uninsurance, minority ethnicity, and limited physician capacity [1]. New Mexico ranks among the lowest in the nation in personal per capita income (47^th^), and among the highest (third) in the proportion of the population below the poverty level (18.4%). Approximately 22% of the population does not have health insurance, and 32 of 33 counties have federally designated Health Professional Shortage Areas and/or Medically Underserved Areas [1]. Recent research has suggested that New Mexico may be struggling to deliver adequate primary care, possibly due to policy changes affecting Medicaid [1]–[5].

As an important component of primary health care, adequate prenatal care is generally considered essential for reducing adverse birth outcomes. Although there are myriad definitions for “adequate” prenatal care, most recommendations seem to indicate that prenatal care should begin during the first trimester and that there should be nine prenatal care visits through 36 weeks of gestation, with an additional visit every week thereafter [6]. Timely and adequate prenatal care has been found to be associated with a reduction in low birth weights, and a corresponding increase in positive birth outcomes. Adequate prenatal care has also been found to be an indicator of utilization of future child health care, including adequate number of well-child visits and up-to-date immunization status [7], [8].

Inadequate prenatal care has been linked to an increased risk of negative birth outcomes. The typical indicators of negative birth outcomes are preterm delivery, defined as a gestational age of less than 37 weeks, and low birth weight (<2500 g) [9]. Compared to those receiving adequate prenatal care, mothers receiving inadequate prenatal care have been shown to have greater than one and one-half times the risk of delivering low birth weight babies [10]. Both gestational age and birth weight have been shown to have strong independent and combined associations with perinatal mortality [11]–[13] and preterm infants are at an increased risk for various sequelae [14]. The financial costs associated with preterm, low birth weight babies are substantial. In California in 2000, nearly six percent of infants weighed <2500 g at birth but accounted for 56.6% of associated hospital costs [15]. In 2001, eight percent of infant births in the United States were diagnosed as preterm birth/low birth weight; however, they consumed 47% of associated hospital costs [16]. Low socioeconomic status, concomitant with untimely and inadequate prenatal care, has been associated with preterm deliveries and low birth weights, making the isolation of specific risk factors problematic [17].

Studies examining barriers to timely and adequate prenatal care have implicated several contributing factors, including unwanted or unplanned pregnancies, reduced access to primary care, and no education beyond high school [18]–[21]. Interestingly, among low-income working women, personal costs associated with prenatal care, including lost wages from missing work, as well as transportation and child care costs, were not associated with untimely or inadequate prenatal care [22].

A study of mothers in California during 1994 and 1995 found that among low-income women who were eligible for the state's Medicaid program (Medi-Cal) and who were not receiving prenatal care, 40% were uninsured. Possible explanations for pregnant women remaining uninsured when eligible for the state's Medicaid program included administrative barriers, undocumented immigrant status, and fear of repercussions associated with substance abuse [21], [23]. In New Mexico, as in most states, low-income pregnant women and mothers with young children are eligible for Medicaid. Access to prenatal care by low-income mothers should, therefore, not be affected by Medicaid eligibility.

The objective of the present research is to compare prenatal care utilization between higher- and low-income areas of New Mexico. A reasonable expectation based on previous research about the impacts of Medicaid reform on primary care in New Mexico [1] is that mothers residing in low-income areas of the state will experience a comparatively lower level of prenatal care.

## Materials and Methods

The State Center for Health Statistics of the New Mexico Department of Health provided annual birth records from 1989 to 1999 for all (n = 31) residential zip codes in the state of New Mexico. The data were originally collected as part of a larger study examining the effects of implementation of Medicaid managed care in 1997 [1]. The Institutional Review Boards of the Health Sciences Center and the general campus of the University of New Mexico approved the methods used in this research. Written consent was not needed for this research because it was based on publically available records without unique identifiers. Because the mother's income level is not recorded on the birth records, births were parsed into higher- and low-income groups based on the published median family income of the mothers' residential zip codes. A family is defined by the US Census Bureau as consisting of the householder and at least one other person living in the household who is related to the householder by birth, marriage, or adoption. Estimates of median family income for each zip code were derived from data published online by the US Census Bureau for the 1990 census. Low-income zip codes were defined as having a yearly median family income of less than 185% of the 1998 federal poverty level of $17,650, or a minimum of $32,652. This corresponded with eligibility for the Medicaid Family Planning and Pregnancy program. Those zip codes with median family incomes greater than $32,652 were classified as higher income.

Prenatal care indicators used in the study included the number of prenatal care visits and the first month of prenatal care. We did not take into account gestational age at birth when calculating the mean number of prenatal care visits. Because we were interested in potential differences between income groupings for both of these aspects of utilization, we did not use an aggregated index of prenatal care adequacy or utilization [6]. Birth outcome indicators included low birth weight, premature birth, and births linked with death certificates. Identification of premature births was based on an estimated clinical gestational age of less than 36 weeks. An infant with a reported weight <2500 grams was considered to have a low birth weight, while infants with birth weights ≥2500 grams were considered to have normal birth weights.

The effect of income grouping on birth outcomes listed on the birth records was estimated using odds ratios and Fisher's exact tests. We used Satterthwaite *t*-tests for unequal sample variances [24] to examine differences between higher- and low-income samples for the mean month of pregnancy when the mother first sought prenatal care, and the mean number of prenatal care visits during pregnancy. The association between adverse birth outcomes and prenatal care indicators irrespective of income area was estimated using nonparametric Spearman correlation. A significance level of α = 0.05 was used for all tests.

## Results

On average, mothers from low-income areas of New Mexico started their prenatal care significantly later in their pregnancies than did mothers from higher-income areas between 1989 and 1999 (p<0.01) (Table 1). Mothers from low-income areas also had significantly fewer prenatal visits between 1989 and 1997 (p<0.0001), but not between 1998 and 1999 (Table 2). Not surprisingly, there was a significant negative correlation between the mean number of prenatal care visits and the mean first month of prenatal care (r_s_ = −0.830, p<0.001). The percentage of mothers giving birth to low-weight babies ranged from 7.34% to 8.40% for mothers from low-income area, and 6.12% to 9.30% for mothers from higher-income areas over the 10 year period. Fisher's exact comparisons failed to reveal any significant differences between income groupings in the proportion of mothers giving birth to low-weight babies (results not shown).

**Table 1:** Comparison of the mean month of first prenatal care visit for mothers of low- and high-income areas of New Mexico.

Year	Income	N	Mean	SD	t	p-value
1989	Low	8081	3.65	2.06	8.73	<0.0001
	Higher	757	3.06	1.75		
1990	Low	8463	3.67	2.01	9.24	<0.0001
	Higher	784	3.08	1.68		
1991	Low	8752	3.59	1.95	8.55	<0.0001
	Higher	807	3.04	1.73		
1992	Low	8863	3.38	1.90	6.64	<0.0001
	Higher	749	2.96	1.62		
1993	Low	8817	3.27	1.84	6.81	<0.0001
	Higher	759	2.89	1.43		
1994	Low	8502	3.19	1.81	6.80	<0.0001
	Higher	845	2.82	1.51		
1995	Low	8074	3.10	1.82	6.59	<0.0001
	Higher	963	2.76	1.48		
1996	Low	8351	3.13	1.83	11.15	<0.0001
	Higher	850	2.55	1.41		
1997	Low	7744	3.07	1.82	7.11	<0.0001
	Higher	959	2.69	1.53		
1998	Low	7647	3.17	1.90	6.38	<0.0001
	Higher	1382	2.86	1.60		
1999	Low	7399	3.15	1.97	3.43	0.001
	Higher	1454	2.98	1.70		

**Table 2:** Comparison of the mean number of prenatal care visits for mothers from low- and high-income areas of New Mexico.

Year	Income	N	Mean	SD	t	p-value
1989	Low	8099	8.99	4.28	12.43	<0.0001
	Higher	758	11.41	5.19		
1990	Low	8414	9.09	4.18	10.18	<0.0001
	Higher	777	10.89	4.77		
1991	Low	8685	9.18	4.07	11.82	<0.0001
	Higher	801	11.19	4.65		
1992	Low	8901	9.27	4.22	8.32	<0.0001
	Higher	745	10.59	4.15		
1993	Low	8795	9.60	4.20	9.58	<0.0001
	Higher	762	11.06	4.40		
1994	Low	8472	9.65	4.01	9.84	<0.0001
	Higher	850	11.00	3.78		
1995	Low	8066	9.65	4.21	9.80	<0.0001
	Higher	962	11.10	4.33		
1996	Low	8313	9.65	4.09	13.83	<0.0001
	Higher	853	12.10	5.00		
1997	Low	7739	9.63	4.18	9.78	<0.0001
	Higher	982	11.23	4.88		
1998	Low	7663	9.52	4.25	1.46	0.144
	Higher	1377	9.70	4.25		
1999	Low	7510	9.54	4.16	0.20	0.843
	Higher	1478	9.51	4.01		

Significant differences between income groups were detected for the proportion of mothers giving birth to premature babies early in the time period assessed (1989 & 1990, p≤0.01), but these differences narrowed during later years, becoming nonsignificant (Table 3). The percentage of births linked with death certificates (perinatal death) ranged from 0.48% to 0.92% for mothers from low-income areas, and 0.19% to 1.02% for mothers from higher-income areas over the 10 year period. No significant differences emerged between low- and higher-income groups in the percentage of perinatal death rates over the 10 year period (results not shown).

**Table 3:** Comparison of the proportion of mothers from low- and high-income areas giving birth to premature babies in New Mexico.

Year	Low Income		Higher Income		Fisher's Exact p	OR	(95% CI)
	%	Total births	%	Total births			
1989	10.59	8479	7.72	803	**0.01**	1.41	(1.08,1.84)
1990	11.08	8785	7.35	830	**0.00**	1.56	(1.19, 2.04)
1991	4.64	8971	4.92	833	0.67	0.93	(0.67, 1.29)
1992	4.60	9135	4.57	788	1.00	0.99	(0.70, 1.41)
1993	4.44	9002	5.34	787	0.24	0.82	(0.59, 1.13)
1994	4.72	8695	4.66	880	1.00	1.00	(0.72, 1.39)
1995	5.03	8369	5.75	1008	0.32	0.86	(0.65, 1.14)
1996	4.89	8613	6.03	879	0.14	0.79	(0.59, 1.06)
1997	4.85	8104	3.52	1022	0.06	1.38	(0.98, 1.95)
1998	5.00	7960	6.07	1433	0.09	0.81	(0.64, 1.03)
1999	4.94	7891	4.87	1539	0.95	1.01	(0.78, 1.30)

OR refers to Odds Ratio.

Irrespective of income area, there were not significant correlations between the mean number of prenatal care visits and the percentage of low-weight babies (r_s_ = 0.069, p = 0.762), the percentage of premature babies (r_s_ = 0.051, p = 0.820), or the percentage of births linked with death certificates (r_s_ = −0.104, p = 0.662). Similarly, there were not significant correlations between the mean first month of prenatal care and the percentage of low-weight babies (r_s_ = −0.095, p = 0.676), the percentage of premature babies (r_s_ = 0.040, p = 0.859), or the percentage of births linked with death certificates (r_s_ = 0.151, p = 0.524).

## Discussion

The results of our analysis indicate significant differences in prenatal care utilization by mothers from low- and higher-income areas. Mothers from low-income areas had fewer prenatal care visits and initiated prenatal care later than mothers from higher-income areas. Despite this disparity in prenatal care, there were only minor differences between income groupings in the relative occurrence of negative birth outcomes. This finding suggests that despite reduced utilization mothers from low-income areas of New Mexico are either receiving an adequate level of prenatal care, or that small—though statistically significant—differences in prenatal care have exerted little direct impact on birth outcomes. Our results also indicate that there was not a significant correlation between prenatal care indicators and adverse birth outcomes irrespective of income area. These findings are generally consistent with critical discourse questioning the efficacy of current prenatal care recommendations in the United States [25]–[28]. Specifically, our study suggests that lower utilization of prenatal care, albeit only marginally so, does not increase adverse birth outcomes for mothers from low income areas of New Mexico. While we do not mean to suggest there are not important benefits associated with prenatal care, our study does contribute to a growing literature questioning whether adequate prenatal care utilization as it is currently defined is the primary driver of birth outcomes.

The reasons for the observed disparities in prenatal care are unclear. Cost, language, and transportation are typically considered the primary barriers to preventive care. Our previous research in New Mexico indicated that difficulty in securing a primary care physician (PCP), cost of services, and transportation likely represented barriers to primary care for low-income residents, both uninsured and Medicaid eligible [1]. Among these barriers, not having a PCP was the most commonly cited barrier to primary care by the uninsured (51.8%), followed closely by cost (48.9%) [1]. In addition to these barriers, research also suggested that health policy changes affecting Medicaid may have exacerbated difficulties in securing a PCP [2]–[4]. Although our present study could not clarify the impact of not having a PCP on the level of prenatal care received, lack of a PCP clearly has affected the availability of similar preventive services for low-income patients.

### Limitations

Our study had several important limitations. Because the mothers' income was not listed on the birth records, we were not able to examine directly the effect of income on prenatal care and birth outcomes. Because our study was ecological in nature, we could not identify the specific reasons for the observed differences in prenatal care between our two income groups. Ecological studies can be misleading because variability at the individual level is lost by using group averages. As such, there is the risk of committing the ecological fallacy, where data are analyzed at the group level but inferences are made at the individual level [29], [30]. We emphasize that we cannot make inferences about prenatal care at the individual level, but rather only at the group level, which for this study was defined by geographic area. Factors not examined in the present research such as rurality and cultural practices may have contributed to the observed disparities in prenatal care. Finally, because New Mexico is a comparatively poor state with only a limited number of high-income geographic areas, sample sizes for the two income groupings were necessarily imbalanced.

In conclusion, our results revealed disparities in prenatal care, with pregnant mothers from low-income areas experiencing reduced utilization of prenatal care relative to mothers from higher-income areas. Although significant disparities in prenatal care utilization were detected, these disparities were not associated with concomitant differences in adverse birth outcomes, suggesting that pregnant mothers from low-income areas received an adequate level of prenatal care relative to mothers from high-income areas, or that a reduced utilization of prenatal care does not exert a substantial direct effect on adverse outcome.
